# Fibropapillomatosis in Green Sea Turtles (*Chelonia mydas*): Etiology, Pathology, Diagnostic Challenges, and Rehabilitation Management

**DOI:** 10.3390/ani16121906

**Published:** 2026-06-19

**Authors:** Manuela Tripepi, Ellianna Ruggeri, Ahmad Arfan, Emily Valenzuela, Isabella Vitales

**Affiliations:** Department of Biological and Chemical Sciences, College of Life Sciences, Jefferson University, Philadelphia, PA 19144, USA; ellianna.ruggeri@students.jefferson.edu (E.R.); ahmad.arfan@students.jefferson.edu (A.A.); emily.valenzuela@students.jefferson.edu (E.V.); isabella.vitales@students.jefferson.edu (I.V.)

**Keywords:** green sea turtles, fibropapilloma, herpesvirus, ChHV5, wildlife pathology, rehabilitation, disease ecology

## Abstract

Fibropapillomatosis (FP) is a disease that threatens the health and safety of sea turtles globally, with green sea turtles having the highest FP prevalence. FP is associated with Chelonid alphaherpesvirus 5, but the primary etiological agent remains unknown as expression and severity of the virus are influenced by host susceptibility, immunological status, development of epithelial lesions, and environmental factors. This review explores current understanding of FP in green sea turtles, focusing on etiology, pathological features, diagnostic approaches, and rehabilitation strategies. Emphasis is placed on the compounded nature of the disease, integrating factors that influence disease expression. Screening challenges are examined through the limitations of clinical, histological, and molecular methods, particularly in distinguishing latent from active infection. Rehabilitation practices, including surgical intervention and long-term supportive care, are evaluated in the context of treatment outcomes and recurrence risk. Collectively, the evidence supports the interpretation of FP as a disease shaped by host–pathogen–ecology interactions. Improved integration of diagnostic tools and greater focus on natural drivers are essential for advancing understanding of the disease and informing conservation and management efforts.

## Simple Summary

Fibropapillomatosis (FP) is a neoplastic disease that affects sea turtle species worldwide, with green sea turtles (*Chelonia mydas*) among the most affected. The disease is characterized by benign fibroepithelial tumors that primarily develop on soft tissues, including the flippers, neck, periocular regions and in some cases affect internal organs. These tumors may also extend onto or occur on keratinized surfaces such as the carapace and plastron. Depending on their size and anatomical distribution, tumors may impair locomotion, vision, feeding, digestion, and overall physiological function. FP is associated with the virus Chelonid alphaherpesvirus 5 (ChHV5); however, it is not confirmed as the sole cause, as the disease is an amalgamation of complex causes, in particular stress and habitat degradation. Pathologically, FP is identified by the development of fibroepithelial tumors that vary in size, shape, texture, and anatomical distribution. Disease testing relies on histological examinations, molecular techniques such as polymerase chain reaction (PCR) and quantitative PCR (qPCR). However, determining etiology is complicated by the presence of ChHV5 in turtles that are otherwise healthy. Rehabilitation tactics, including surgical intervention and long-term monitoring, serve as effective strategies to safely reintroduce these animals back into their natural habitat, although challenges such as tumor regrowth and potential ChHV5 latency stress the need for continued analysis of FP pathogenesis.

## 1. Introduction

Fibropapillomatosis (FP) is a worldwide neoplastic disease documented in all extant sea turtle species, with the highest prevalence in green sea turtles (*Chelonia mydas*) [1,2].

Characterized by the formation of external and internal tumors, FP can hinder movement, degrade and occlude vision, impair feeding and buoyancy, and compromise overall physiological function, rendering it a major wildlife health issue impacting sea turtles globally [1,3]. The disease has been reported throughout the Atlantic, Pacific, and Indian Oceans, as well as the Caribbean and Mediterranean regions, and has been documented in sea turtle populations across North America, South America, Africa, Asia, and Australia [1,4,5,6]. Although FP is most visibly characterized by external cutaneous and ocular tumors, visceral tumors have also been reported in the lungs, kidneys, heart, liver, spleen, gastrointestinal tract, and musculoskeletal tissues, where they can impair organ function and contribute to debilitation or mortality in severely affected turtles [4,7,8]. The widespread geographic distribution and potentially severe health consequences of FP highlight its significance as a global conservation concern [1,5,9].

Given its rising incidence and consequences for individual fitness and population health, FP has emerged as a significant concern in animal pathology, disease ecology, and marine conservation medicine, with emerging reports documenting its presence in previously underreported regions [10], including the Caribbean [11].

FP is strongly linked to Chelonid alphaherpesvirus 5 (ChHV5) [6,12,13,14,15]. This herpesvirus has been consistently found in the tumors of affected turtles, as shown by molecular techniques like polymerase chain reaction (PCR) and quantitative PCR (qPCR). Early research by Lackovich et al. showed the presence of herpesvirus DNA in FP tumors, and the work by Quackenbush et al. supported this association by amplifying viral DNA in fibropapilloma samples [16,17]. However, detection of ChHV5 in clinically healthy turtles suggests that the virus alone does not cause the disease [3,18]. The virus might stay dormant and other determinants likely contribute to the disease’s development [19].

Current investigations corroborate the perspective that FP represents a multifaceted disease, rather than a straightforward neoplasm instigated solely by a virus. The expression and intensity of the disease are substantially influenced by host susceptibility, immunological status, developmental phase, and environmental factors [20,21]. Juvenile and subadult green turtles appear to be at heightened risk, potentially attributable to their habitat, age, or the efficacy of their immune responses [19]. Furthermore, the prevalence of FP has been linked to compromised coastal ecosystems, human-induced disturbances [22], and exposure to pollutants, with region-specific studies documenting spatial variation in contaminant exposure in habitats utilized by sea turtles [23], and linking habitat use with disease occurrence [11,20,24].

Heavy metals, persistent organic pollutants, and toxins associated with harmful algal blooms are linked to FP [25]. Keller et al. (2004) [25] documented contaminant levels in sea turtles from areas impacted by FP, and Perrault et al. (2017) subsequently investigated the relationships between physiological stress, toxicological exposure, and the overall health of sea turtles [26]. These observations support the hypothesis that FP may arise from the interplay of infectious exposure and environmentally induced immunosuppression or chronic physiological stress [20,26]. Consequently, these trends position FP within the wider context of conservation medicine, where wildlife disease is conceptualized because of both host–pathogen interactions and ecosystem health.

From a pathological standpoint, FP is characterized by the development of fibroepithelial tumors that vary widely in size, anatomical distribution, and external appearance. These lesions may present as smooth or rugose masses occurring either singly or as multifocal lesions, and can develop on the skin, around the eyes, on the flippers, in the mouth, and in internal organs (including lungs, kidneys, heart, liver, spleen, and the gastrointestinal tract) [1,27,28,29]. Histologically, fibropapillomas are often linked to epidermal thickening, projections from the epidermis, increased fibroblast activity, ballooning degeneration, and various degrees of connective tissue remodeling [18,30,31]. Work et al. (2004) described the different forms of these lesions in green turtles, documenting both external and internal tumors, including fibropapillomas, myxofibromas, fibromas, papillomas, and low-grade fibrosarcomas, while Norton et al. also reported internal fibromatous lesions in affected turtles [30,32].

Although many tumors associated with FP appear benign under a microscope, they can have serious clinical effects. These effects are particularly concerning when the tumors block vision, impair feeding efficiency, interfere with swimming, or affect the function of internal organs [1,28].

Because gross appearance alone may not accurately reflect disease burden, the diagnosis of FP increasingly relies on a comprehensive approach that includes clinical examination, histology, and molecular testing [1,28,30]. Histological examination is crucial for confirming lesion classification and identifying proliferative changes in epithelial and dermal tissues, whereas molecular methodologies provide evidence of viral association [18,30,31]. PCR-based studies have shown ChHV5 DNA in tumors and clinically normal tissues, indicating the possibility of viral latency [17,18,27]. Furthermore, in situ hybridization has detected viral transcriptional activity inside acanthotic epithelial cells of fibropapillomas, suggesting that active viral expression may be localized to specific tissue compartments rather than uniformly dispersed throughout the lesion [33]. These findings together emphasize the imperative of contextualizing molecular discoveries within pathological and clinical data, rather than evaluating them in isolation [1,18].

FP poses considerable difficulties in wildlife rehabilitation and conservation efforts, extending beyond the initial diagnostic phase. Turtles afflicted by this condition often present with substantial tumor loads, buoyancy issues, compromised physical condition, and opportunistic infections, thereby necessitating extensive supportive care and surgical interventions [1,8,29]. Clinical rehabilitation protocols typically encompass fluid administration, nutritional supplementation, wound management, and the surgical removal of obstructive tumors, especially those impacting the ocular or oral regions [1,12,34].

Page-Karjian et al. (2014) reported outcomes and recurrence patterns in rehabilitated green turtles with FP, emphasizing that surgery can improve function and quality of life, but recurrence is common and long-term prognosis remains variable [8]. Additional studies have also shown that rehabilitation outcomes depend not only on tumor removal, but also on recovery of physiological stability, feeding ability, and normal locomotor function prior to release [8,35]. As a result, FP should be viewed not only as a pathological challenge, but also as a persistent management issue with direct implications for animal welfare and conservation outcomes.

FP, when considered holistically, serves as a significant model for elucidating the interplay of infectious disease, pathological processes, physical milieu of habitation, and host vulnerability within free-ranging wildlife populations. Several reviews have examined FP in sea turtles, focusing primarily on the association between ChHV5 and disease occurrence. This review adopts a broader, more integrative perspective by reporting on current knowledge on disease ecology, pathology, lesion classification, diagnostic challenges, molecular diagnostics, rehabilitation management, recurrence patterns, differential diagnosis, concurrent conditions, and conservation implications. Emphasis is placed on the interaction between host susceptibility, environmental stressors, and viral infection, reflecting the growing recognition that FP represents a multifactorial disease process rather than a simple virus-associated neoplasm. This report consolidates the existing and latest research concerning the causes, pathological manifestations, diagnostic screening methodologies, and rehabilitation strategies associated with FP in green sea turtles. By integrating these perspectives and recent advances across related disciplines, this report establishes a more comprehensive and up-to-date understanding of disease progression, facilitates enhanced diagnostic evaluation and wildlife health management within affected populations, and identifies priorities for future research, clinical management, and conservation efforts.

## 2. Review Methodology

A narrative literature review was performed using the PubMed^®^, Scopus^®^, and Google Scholar^®^ databases to identify peer-reviewed literature on FP in sea turtles, with special focus on green sea turtles. Articles published in English until February 2026 were included. Studies discussing FP etiology, ChHV5, histopathology, molecular diagnostics, rehabilitation management, environmental risk factors, and conservation implications were prioritized.

Search terms were used individually and in combination and included: “fibropapillomatosis”, “green sea turtle”, “*Chelonia mydas*”, “Chelonid alphaherpesvirus 5”, “ChHV5”, “sea turtle rehabilitation”, “fibropapilloma histopathology”, “marine turtle disease”, “molecular diagnostics”, “PCR”, “qPCR”, “environmental stressors”, “tumor regression”, and “wildlife pathology”.

Additional references were identified through citation tracking of relevant articles and review papers. Publications were selected based on their scientific relevance, contribution to the understanding of FP pathogenesis and clinical significance, and applicability to rehabilitation and conservation management. Both original research articles and relevant review papers were included to provide a comprehensive overview of the current understanding of FP in sea turtles.

## 3. Etiology and Multifactorial Considerations

While all sea turtle species are impacted by FP, green sea turtles are the most frequently affected [21].

As stated before, FP has been closely linked to ChHV5, which has been identified in tumor tissues by molecular techniques including PCR and qPCR [16,36,37]. ChHV5 is regularly associated with FP but has not been conclusively identified as the exclusive causative agent of the disease [15].

The virus has been identified in clinically healthy turtles, while turtles with FP may exhibit variable viral detection depending on tissue type, sampling method, or stage of infection [3,38]. These findings suggest that the presence of ChHV5 alone is insufficient to account for disease manifestation.

The transmission of ChHV5 is believed to occur via several mechanisms, including direct contact, viral shedding into the water column, and possibly mechanical transfer by marine leeches [3,15,39,40]. The exact timing of infection and disease development is ambiguous. Nevertheless, FP is predominantly detected in juvenile and subadult turtles, potentially indicating age-related variations in immune function, and habitat utilization or exposure [19].

**Figure 1:**
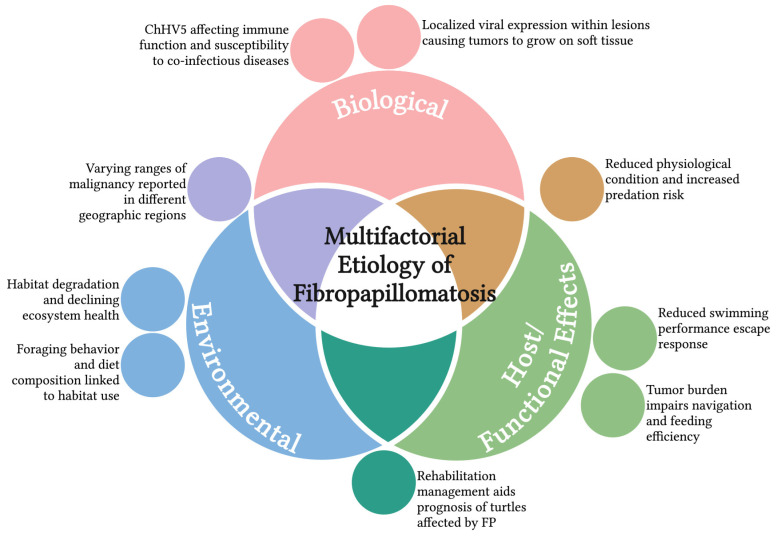
The Multifactorial Etiology of Fibropapillomatosis. This schematic highlights the interacting biological, environmental, and host factors contributing to FP. Biological drivers include ChHV5 infection and immune modulation; environmental influences include habitat degradation, diet, and geographic variation; and host effects include reduced condition, impaired swimming, and decreased feeding efficiency. Rehabilitation and clinical management may further influence disease outcomes, reflecting the complex nature of FP.

Current research indicates that FP is a complex disease influenced by the interplay of viral infection, host susceptibility, immunological status, and ecosystemic stresses [20,21] (Figure 1). Habitat degradation, coastal urbanization, and pollution have all been linked to heightened prevalence of FP in impacted areas. Exposure to contaminants, including persistent organic pollutants, heavy metals, and toxins from harmful algal blooms, may induce immunosuppression or physiological stress, potentially promoting tumor development or progression [24,25,26,41,42].

Turtles affected by FP may encounter comorbidities with the cofactors, such as secondary infections, diminished body condition, compromised motility, and buoyancy irregularities, all of which can exacerbate illness severity and survival rates [20,21].

These findings highlight the importance of contextualizing molecular results within clinical and pathological findings.

## 4. Clinical Presentation and Gross Lesions

Sea turtles affected by FP present a spectrum of clinical manifestations, contingent upon tumor burden, anatomical localization, and concurrent internal pathologies. In a clinical context, FP can compromise critical physiological functions, such as feeding, locomotion, vision, buoyancy regulation, and predator evasion. Large tumors or those situated in strategic locations can impede swimming efficiency and escape behaviors, consequently increasing vulnerability to predation and reducing overall survival prospects [43].

**Figure 2:**
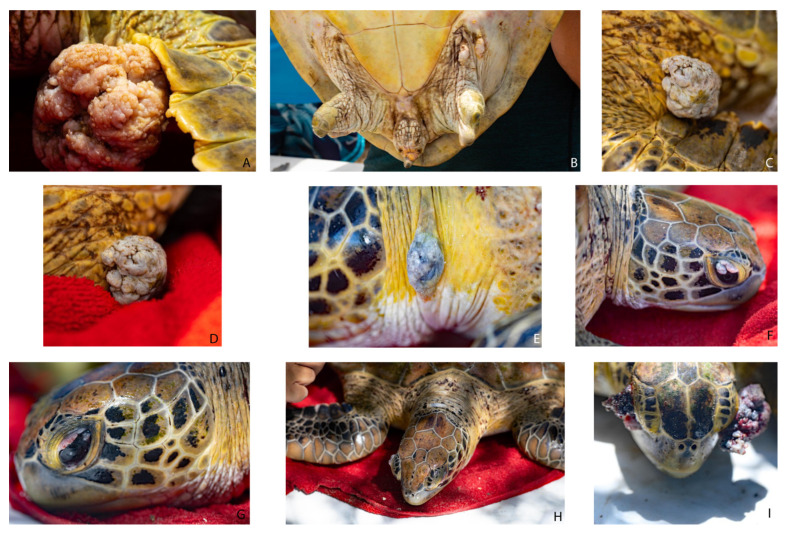
Gross lesions of fibropapillomatosis in green sea turtles caught in Curaçao. Representative external fibropapillomas demonstrating variation in morphology, anatomical distribution, pigmentation, and severity. (**A**–**C**) Large multilobulated rugose fibropapillomas with cauliflower-like architecture adjacent to the plastron and inguinal region. (**D**,**E**) Mildly rugose and smooth nodular fibropapillomas with pale exophytic growth. (**F**–**H**) Periocular fibropapillomas affecting the palpebral margins and surrounding soft tissues. (**I**) Advanced vascularized and ulcerative periocular fibropapilloma with marked vascularization. Tumors varied considerably in size, from less than a cm to ~4 cm, surface texture, and gross appearance. Photographs courtesy of Russ Gooding.

Ocular fibropapillomas, which affect the tissues around the eyes, including the conjunctiva and cornea, can result in visual impairment or blindness and contribute to systemic debilitation [44] (Figure 2). In addition, oral tumors are often detected. A large study of turtle necropsies showed that about 80% of turtles with FP had oral tumors, and more than half had tumors in the glottis [30]. These growths may obstruct the airway, thereby increasing the risk of secondary complications, including pneumonia [30].

The involvement of internal organs is a clinically significant facet of FP. Visceral tumors have been documented in various organs, including the lungs, kidneys, heart, liver, stomach and gastrointestinal tract, including the intestinal mucosa and oropharyngeal regions [30,45,46]. Lesions affecting essential organs can compromise cardiac function, digestion, and buoyancy regulation, diminishing survival rates [7].

Clinical outcomes differ among individuals, especially in rehabilitative contexts where prognosis is significantly affected by tumor burden and systemic involvement [8].

Alterations in immune function are also associated with FP. Studies have demonstrated immunological profile shifts between affected and unaffected turtles. These variations encompass indicators of immunosuppression, reduced lymphocyte activity, and diminished antiviral responses [47,48]. Chronic inflammatory states are commonly observed and may promote tumor persistence and progression. Consequently, these findings suggest that impaired immune surveillance and persistent inflammation substantially influence disease advancement.

FP tumors can appear in various soft tissues, including the flippers, tail, neck, around the eyes, and the groin (Figure 2). These lesions can appear as smooth plaques, papillary, verrucous, nodular, pedunculated, or cauliflower-like growths and may exhibit ulceration, necrosis, or secondary infection [29,49]. Lesions may exhibit considerable variation in pigmentation, ranging from pale white and pink to dark purple or black, and their size can range from a few centimeters to several centimeters in diameter [8,29].

Cutaneous FP is the most prevalent form of the condition, while visceral FP is less common but usually more serious.

## 5. Histopathology and Morphological Diagnosis

**Figure 3:**
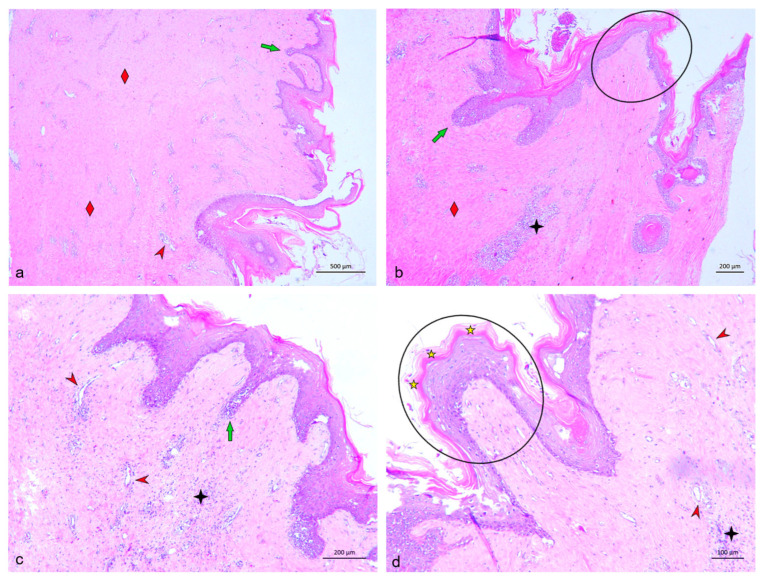
Representative histopathological features of fibropapillomatosis in green sea turtles (*Chelonia mydas*). Samples were fixed in 10% formalin for 48 h and routinely processed for histopathological evaluation. Histologic sections demonstrate dermal connective tissue proliferation (**a**,**b**), neovascularization (**a**,**c**,**d**), inflammatory infiltrates (**b**–**d**), papillomatous acanthosis, edema, and regions of orthokeratotic and parakeratotic hyperkeratosis (**d**). Fibrovascular proliferation (red rhombi), neovascularization (red arrowheads), papillary projections extending into the dermis (green arrows), inflammatory infiltrates (black stars), and parakeratosis (yellow stars) are indicated. Adapted from Pérez et al. (2024) [51].

FP is characterized by the development and progressive growth of fibroepithelial tumors, which vary widely in size, number, and anatomical distribution [27] (Figure 3). These benign growths or fibroepithelial lesions can be used to identify a green sea turtle as FP positive [1]. Globally, FP is characterized by external fibropapillomas and internal fibromas in these animals [27]. Common tumors observed in FP positive green sea turtles are myxofibromas, fibrosarcomas, papillomas, fibromas and fibropapillomas [30,32] (Table 1). Recent efforts have emphasized the importance of establishing standardized terminology and consensus classification systems for fibropapillomatosis-associated lesions in sea turtles [50].

### 5.1. Malignancy of Common Tumors

Although FP is generally associated with histologically benign neoplasms, low-grade fibrosarcomas, a more aggressive lesion, have been observed in green sea turtles, indicating that some cancers associated with fibropapillomatosis can exhibit locally invasive properties [30,32,51].

Significantly, even nonmalignant internal tumors can lead to considerable morbidity by compromising organ function based on their size and anatomical positioning [28]. Consequently, the clinical severity of FP is contingent not only upon tumor histology but also tumor mass, invasiveness, and anatomical distribution. This distinction is important because gross appearance alone may underestimate disease severity, particularly when internal or invasive tumors are present.

### 5.2. Fibropapillomas: Further Characterization

The identification of external fibropapillomas has been used to diagnose infected green sea turtles worldwide [27]. These epithelial lesions often appear on the upper body [53]. To assess how the disease progresses, two classifications, rugose and smooth, have been studied.

Rugose tumors are characterized by a cauliflower-like morphology [30]. Green sea turtle studies at the University of Florida Whitney Laboratory Sea Turtle Hospital in St. Augustine, Florida, confirmed that rugose tumor growth advances more rapidly than smooth tumors [29]. The predominance of smooth tumors has been proposed as a potential indicator of disease regression [29]. Furthermore, fibropapillomas have been categorized as verrucous or fibromatous. Verrucous tumors were characterized by papillary projections of the epidermis, underpinned by a fibrovascular connective tissue matrix with distinctive fibroblasts. Fibromatous tumors comprised a hypercellular dermis consisting of fibroblasts and dermal collagen. Both verrucous and fibromatous tumors exhibited clusters of ballooning, degenerating acanthotic epithelial cells [18]. Ballooning degeneration of cells has been observed in sea turtle species infected with ChHV5. Furthermore, histopathological examination revealed eosinophilic intracellular inclusions associated with ballooning degeneration areas in two juvenile green sea turtles. Electron microscopy confirmed inclusions contained virus-like particles that are morphologically similar to particles belonging to the Herpetoviridae family [54].

In green sea turtles histological diagnosis commonly includes papillary epidermal and dermal hyperplasia, further supporting the proliferative nature of these lesions [31].

### 5.3. Molecular Diagnostics

Molecular approaches have been widely used to detect ChHV5 in sea turtles affected by FP (Table 2). Using quantitative real-time PCR, ChHV5 DNA has been detected in both FP tumors and tissues from clinically healthy turtles, supporting the hypothesis that the virus may persist in dormancy within host populations in locations such as Hawaii, Florida, and Costa Rica [17,18]. For example, 94.4% of samples (17/18 tissues) were PCR-positive for ChHV5 DNA in a study by Duarte et al. (2012) [18]. Similarly, efforts to amplify the DNA polymerase gene of chelonid fibropapilloma-associated herpesvirus (CFPHV) using herpesvirus-specific primers (GTHV1 and GTHV2) identified ChHV5 DNA in 13 of 14 tumor samples [17].

The detection of viral DNA via PCR does not differentiate between active infection and latent viral presence, hence constraining its capacity to directly associate ChHV5 detection with illness manifestation [17].

In addition to PCR methods, in situ hybridization (ISH) has been employed to identify viral nucleic acids within tumor tissues. ISH examination of fibropapillomas from green sea turtles in Puerto Rico revealed ChHV5 mRNA expression in the nuclei of acanthotic epithelial cells, but subepithelial fibrous areas tested negative [33]. The findings indicate that viral activity may be localized inside cellular compartments. Viral transcriptional activity, as opposed to merely the presence of viral DNA, may be crucial for comprehending disease progression [18]. Collectively, these findings suggest that viral pathology must be analyzed in conjunction with histological and clinical data to enhance the evaluation of ChHV5’s involvement in FP development.

### 5.4. Differential Diagnosis

Although FP is often recognized by its characteristic external tumors, lesion appearance alone is not always sufficient for diagnosis. Fibropapillomas can vary considerably in size, shape, texture, and anatomical location, and atypical lesions may resemble other proliferative or inflammatory conditions encountered in sea turtles [46].

A diversity of lesions should be considered when evaluating suspected cases of FP, such as papillomas, fibromas, low-grade fibrosarcomas, inflammatory granulomas, traumatic proliferative lesions, bacterial abscesses, and other cutaneous masses [46,52]. These conditions may resemble the gross appearance of fibropapillomas, while inflammatory, infectious, and neoplastic processes unrelated to ChHV5 infection may mimic the visceral manifestations of FP [46]. FP is often identified based on gross examination, so consideration of these differential diagnoses is important to avoid misclassification. Histopathological examination remains essential for confirming diagnosis and distinguishing FP from other proliferative and neoplastic lesions reported in sea turtles [30,52].

Visceral lesions may not be apparent during routine clinical examination and can represent a variety of inflammatory, infectious, or neoplastic processes. Histopathology remains essential for lesion characterization and differentiation among the tumor types reported in association with FP [30,46]. Therefore, lesions suspected to represent FP should be interpreted cautiously when diagnosis is based solely on gross appearance. Combined clinical, histopathological, and molecular evaluation provides the most reliable approach for diagnostic interpretation [50].

### 5.5. Concurrent Condition

In addition to the characteristic tumors of FP, affected turtles may present with other concurrent conditions that may influence disease severity, prognosis, and rehabilitation outcomes. Secondary bacterial infections may develop in ulcerated or traumatized lesions, while inflammatory changes, parasitic infestations, and other opportunistic conditions may be present in debilitated individuals [8,46,48]. Fungal colonization and secondary wound infections have also been reported in rehabilitating sea turtles and may complicate recovery in the presence of extensive lesions or compromised tissue integrity [55,56].

Other viral infections have also been reported in sea turtles with FP. For example, *Chelonia mydas* papillomavirus 1 has been detected in both tumored and non-tumored green turtles, suggesting that additional viral agents may occur alongside ChHV5 in affected populations [36]. Furthermore, FP-affected turtles frequently exhibit abnormalities in body condition, hematologic parameters, and immune function, indicating that systemic health status may contribute to disease progression and clinical outcome [8,47,48].

Comprehensive evaluation of affected turtles should therefore consider not only tumor burden but also coexisting infectious, parasitic, and systemic conditions that may affect prognosis and rehabilitation outcomes.

### 5.6. Suggested Diagnostic Framework

An ideal approach to diagnosing FP should be tiered and integrated because no single diagnostic method can definitively establish disease status or predict clinical outcome [1,18,30]. Physical examination, body condition scoring, photographic documentation, and evaluation of vision, feeding ability, swimming performance, and buoyancy should be included in the initial evaluation [1,28]. The gross appearance of lesions can provide useful information as a preliminary diagnostic tool but should not be used as a sole basis for diagnosis [1,28]. Histopathological evaluation remains the gold standard for the characterization of lesions and is the essential means for confirming the tumor type, while excluding other proliferative, inflammatory or infectious conditions [18,30,31]. Molecular techniques, such as PCR and qPCR, can demonstrate the association of ChHV5, but the results should be interpreted with caution as viral DNA can also be found in clinically healthy turtles and does not necessarily indicate active disease [17,18]. Diagnostic findings should be interpreted in the context of clinical presentation, distribution of lesions, histopathological findings and assessment for concurrent conditions, if possible [1,18,30]. In severely affected animals, advanced imaging techniques such as radiography, ultrasonography, computed tomography or magnetic resonance imaging may also help to identify visceral involvement [7,30,45,46]. The combination of these approaches may improve diagnostic accuracy, aid clinical decision making, and give a more complete picture of disease severity and prognosis [1,18,30].

## 6. Rehabilitation Management

The rehabilitation of fibropapillomatosis-positive green sea turtles aims to restore physiological stability and functional mobility before their reintroduction into marine habitats. Rehabilitation programs typically involve clinical stabilization, supportive care, nutritional management, treatment of underlying conditions, and assessment of suitability for release [1,56]. Admission to rehabilitation centers typically follows stranding incidents linked to swimming difficulties, vision-obstructing tumors, impaired feeding capabilities, or overall frailty [1,8,29]. The preliminary clinical examination generally encompasses hematologic and biochemical analysis, external tumor burden assessment, and body condition evaluation to determine therapy eligibility and prognosis [8,57]. Individuals exhibiting small exterior tumors typically show enhanced rehabilitation potential compared to those with severe debilitation, significant tumor load, or probable internal involvement [1,8]. Stabilization precedes surgical intervention. Turtles infected with FP commonly exhibit dehydration, anemia, emaciation, and secondary bacterial infections linked to ulcerated tumor surfaces or extended debilitation [1,8]. Consequently, supportive treatment strategies often encompass fluid therapy, antimicrobial medication when clinically warranted, wound management, and regulated nutritional rehabilitation intended to restore metabolic and immunological functions [1,34]. Enhanced nutritional status during captivity has been linked to improved physiological recovery, with instances of partial tumor remission noted in rehabilitative subjects [29,34].

### 6.1. Surgical Rehabilitation Procedures

Surgical excision remains the primary therapeutic approach utilized in FP rehabilitation [1,57]. Fibropapillomas located periocularly or within the oral cavity are often prioritized because of their direct interference with vision, environmental navigation, and feeding [1,30]. Laser and electrosurgical removal methods are commonly implemented to reduce hemorrhage and improve precision while preserving surrounding tissue integrity [12,34,57]. Post-operative improvement in feeding ability, visual function, and overall activity has been reported following removal of obstructive tumors, particularly in turtles with severe periocular or oral involvement [1,34]. The severity, anatomical distribution, and functional impact of fibropapillomas can influence both surgical planning and rehabilitation outcomes. Tumors affecting the periocular region, oral cavity, or flippers often present greater clinical challenges because they may impair vision, feeding, locomotion, and normal behavioral functions. In cases involving extensive tumor burdens, complete removal may require careful prioritization of lesions that most significantly compromise quality of life and survival. The restoration of normal feeding behavior, mobility, and overall activity following tumor excision is therefore an important indicator of surgical success and rehabilitation progress [1,28,30,34]. Despite successful excision, recurrence of fibropapillomas remains frequently documented [57]. Tumor regrowth has been reported in approximately 50% of surgically treated rehabilitated green sea turtles, supporting the interpretation that surgical removal reduces tumor burden without eliminating latent ChHV5 infection within host tissues [57].

### 6.2. Environmental and Supportive Rehabilitation

Environmental conditions maintained during rehabilitation may also influence recovery outcomes in fibropapillomatosis-affected sea turtles [1,58]. Water quality is carefully regulated in rehabilitation facilities because unstable salinity, temperature, or hygiene conditions may increase physiological stress in already compromised individuals [1,8]. Housing conditions are likewise managed to reduce disturbance while turtles recover strength, normal swimming behavior, and feeding activity. Handling is generally limited to essential medical procedures, as repeated disturbance may suppress recovery and delay healing in debilitated animals [1]. Stable rehabilitation conditions may support improved physiological recovery during captivity [8,58].

Exposure to natural sunlight or ultraviolet (UV) radiation has also been investigated during sea turtle rehabilitation [58]. Ultraviolet radiation contributes to vitamin D synthesis, which plays an important role in calcium regulation, metabolism, and immune function [58]. Rehabilitating green sea turtles exposed to greater levels of natural sunlight demonstrated significantly higher circulating vitamin D concentrations than turtles maintained under lower UV exposure conditions, suggesting that enclosure design permitting sunlight access may support physiological recovery during captivity [58]. These findings suggest that location management during rehabilitation may influence physiological recovery and overall health status. Rehabilitation periods often extend for several months, allowing recovery of body condition and muscular strength prior to release evaluation [1,57].

### 6.3. Post-Rehabilitation Assessment and Release

Successful rehabilitation requires restoring functional abilities, not just removing the tumor [1,59]. Standardized release criteria often incorporate behavioral, clinical, and physiological assessments to improve post-release survival outcomes [8]. Animals exhibiting impaired locomotion or an inability to forage independently are considered poor candidates for release, as these deficits significantly reduce their likelihood of survival in the wild [1,8]. Satellite telemetry studies of rehabilitated green sea turtles have demonstrated that released turtles can resume directed locomotion, feeding activities, and migratory behaviors upon reintroduction in the wild [35,59]. Before an animal is released, its swimming endurance, buoyancy control, normal diving behavior, and ability to feed independently are evaluated. Animals exhibiting impaired locomotion or impaired independent foraging ability are generally excluded from release because these deficits are associated with reduced post-release survival [1,57].

### 6.4. Cost and Resource Demands of Sea Turtle Rehabilitation

Sea turtle rehabilitation requires extensive resources and high costs, including infrastructure, staff, and long-term care. Rehabilitation is inherently resource-intensive and places substantial demands on program operators, with outcomes varying and successful release dependent on factors such as disease severity, duration of care, and individual response to treatment [8,60]. Facilities must maintain proper water quality, have access to specialized medical equipment, and employ qualified staff, in addition to the direct costs of treating each turtle [61]. The process may also involve surgical procedures, prolonged nutritional support, and regulated living conditions, which further contribute to overall cost. At the programmatic level, the creation and maintenance of rehabilitation centers depend on ongoing funding and resource allocation [62,63]. Estimated rehabilitation costs may reach several thousand U.S. dollars per individual [63,64]. The total cost is not fixed and can vary widely depending on disease severity, treatment duration, facility resources, and required medical intervention.

### 6.5. Proposed Rehabilitation Framework

Successful rehabilitation of sea turtles with FP requires more than tumor removal and should focus on restoring overall health and normal physiological function [1,8,35]. Protocols differ across institutions, but a structured framework may lead to greater consistency in patient management and facilitate comparison of treatment outcomes. A functional rehabilitation workflow might consist of (1) intake examination and stabilization, (2) assessment of tumor burden and systemic health, (3) supportive care including hydration and nutritional supplementation, (4) surgical removal of clinically significant tumors, (5) wound management and monitoring for recurrence, (6) assessment of swimming ability, buoyancy control, and feeding performance, and (7) post-release monitoring when possible. Treatment of dehydration, malnutrition, secondary infections, and other conditions that may compromise recovery should be included in the initial management [1,8,34]. The primary treatment for tumors that compromise vision, feeding, locomotion or respiration is surgical excision, with recurrence a possible complication [8,29,34]. The decisions about the release should not be based solely on tumor regression, but also on body condition, normal behavior, swimming ability, and the ability to forage independently [8,35]. Long-term monitoring of rehabilitated turtles can provide valuable information about recurrence and survival following release [8,35]. More standardized rehabilitation records and outcome reporting may enable comparisons across facilities and help develop evidence-based management guidelines for FP.

## 7. Research Priorities and Future Directions

Despite decades of study, basic questions regarding the pathogenesis and progression of FP remain unanswered [3,15,20,21]. ChHV5 is invariably associated with FP lesions, but the mechanisms underlying the interactions among viral infection, host susceptibility, immune function, and environmental stressors in initiating tumor development remain poorly understood [3,15,20,21]. Additional longitudinal studies are necessary to differentiate viral exposure from disease causation better and to identify factors that trigger progression from latent infection to clinically apparent disease [17,18,20].

Standardization of systems for lesion classification, severity scoring, diagnostic criteria, and reporting of rehabilitation outcomes should be the aim of future research [50]. Increased standardization would allow comparisons across studies, rehabilitation facilities, and geographic regions, thereby increasing the reproducibility of future studies. Further work is also needed to better characterize the prevalence of visceral disease, risk factors for recurrence, and long-term survival following rehabilitation and release [8,29,30,35].

Emerging molecular technologies such as transcriptomics, environmental DNA surveillance, metagenomics, and biomarker discovery may provide novel insights into disease pathogenesis and improve early detection capabilities [15,36,37]. Similarly, further research on the effects of habitat degradation, pollution, habitat changes, and climate-driven harmful algal blooms will enhance understanding of the roles these factors play in disease emergence and dynamics [24,25,26,41,42].

From a conservation perspective, combining disease surveillance with ecosystem monitoring programs could provide a more holistic picture of FP dynamics at the individual and population levels [20,21,24]. Improved collaboration among rehabilitation facilities, researchers, veterinarians, and conservation organizations will be essential to developing evidence-based management strategies that promote the long-term recovery and conservation of affected sea turtle populations.

## 8. Conclusions

FP in green sea turtles is a multifactorial disease that transcends a mere virus–tumor association. While robust correlations with Chelonid alphaherpesvirus 5 (ChHV5) have been consistently established, current evidence supports a more comprehensive disease model in which viral presence, host susceptibility, immune function, and regional stressors collectively modulate disease manifestation and progression. The widespread detection of ChHV5 in both symptomatic and asymptomatic individuals underscore the need to examine viral dormancy and cofactors to elucidate FP pathogenesis.

Recent advances in disease assessment methods, such as histopathology and molecular techniques, have made it easier to identify and characterize FP. However, distinguishing an active infection from a dormant virus remains difficult, underscoring the need for clinical frameworks that integrate clinical, pathological, and molecular data. From a management standpoint, rehabilitation initiatives that include surgical intervention and supportive care are essential for enhancing individual outcomes; however, elevated recurrence rates underscore that existing treatments are not curative.

The rising incidence of FP in areas affected by anthropogenic stressors underscores its significance in the broader framework of conservation medicine. Environmental degradation, pollution, and ecosystem imbalance likely make diseases more common and severe, making FP both a wildlife health problem and a sign of ecosystem health. Future research should focus on longitudinal studies, standardized diagnostic criteria, and the examination of these determinants to enhance the understanding of disease dynamics.

To more effectively address fibropapillomatosis, efforts across pathology, virology, ecology, and conservation are essential. Such a multidisciplinary approach is critical not only for improving the health outcomes of affected turtles, but also for informing targeted, evidence-based conservation strategies in marine ecosystems that are increasingly under environmental stress.

## Figures and Tables

**Table 1:** Common Tumors associated with Fibropapillomatosis (FP).

Tumor Type	Malignancy	Histological/Morphological Features	Reported Anatomical Locations	Geographic Region Reported	References
**Fibropapillomas**	Benign	Exophytic lesions categorized as rugose (papilla-like projections) or smooth	Oropharyngeal region, Front Flippers, Stomach, Intestinal mucosa	West Africa Hawaiian Islands	[18,28,29,30]
**Myxofibromas**	Benign	Loose to dense collagen bundles with varying numbers of pleomorphic fibroblasts	Spleen, intestines	Hawaiian Islands Florida	[30,32]
**Fibromas**	Benign	Dense collagen bundles with varying quantities of pleomorphic fibroblasts	Lungs, kidneys, musculoskeletal system	Hawaiian Islands	[30]
**Fibrosarcomas**	Low-grade malignant	Invasion of bone tissue	Heart: right atrium, bone associated tissue	Hawaiian Islands	Work et al., 2004 [30]
**Papillomas**	Benign	Early-stage epidermal proliferative lesions	Epidermis, inner eyelids	Hawaiian Islands Florida	[33,52]

**Table 2:** Diagnostic Methods Used in the Detection and Characterization of Fibropapillomatosis in Sea Turtles.

Method	What It Detects	Strengths	Limitations	Ability to Detect Active Infection	Interpretation Considerations	References
**Clinical Examination**	External tumor presence, distribution, severity	Non-invasive, rapid, field-applicable	Cannot detect internal disease; subjective severity scoring	No	May underestimate total disease burden, especially with visceral FP	[1,28]
**Histopathology**	Tissue architecture, cellular changes, tumor classification	Gold standard for lesion characterization; distinguishes tumor types	Requires biopsy; invasive; lab-dependent	No	Confirms lesion morphology but not viral causation	[18,30,31]
**PCR (Conventional)**	Presence of ChHV5 DNA	Sensitive; widely used; confirms viral association	Cannot distinguish latent vs. active infection	No	Positive result cannot confirm causation of disease	[17,18]
**qPCR (Quantitative PCR)**	Viral DNA load (relative quantification)	Highly sensitive; allows viral load comparison	Cannot confirm active viral replication	Limited/Indirect	Viral load may not directly correlate with disease severity	[17,18]
**In Situ Hybridization (ISH)**	Localization of viral nucleic acids within tissue	Identifies sites of active viral transcription	Technically complex; less widely available	Yes	Helps differentiate active viral expression from latent presence	[33]
**Imaging (Radiography/Ultrasound)**	Internal tumors, organ involvement	Non-invasive evaluation of visceral disease	Limited sensitivity depending on modality and tumor size	No	Important for detecting internal FP not visible externally	[30,45]
**Hematology/Biochemistry**	Physiological status (e.g., anemia, immune function, stress indicators)	Supports clinical assessment; useful for prognosis	Not specific for FP	No	Useful for evaluating disease severity, immune status, and clinical stability	[8,48]

## Data Availability

No new data were created or analyzed in this manuscript. Data sharing is not applicable to this article.

## Abbreviations

The following abbreviations are used in this manuscript:

FP	Fibropapillomatosis
ChHV5	Chelonid alphaherpesvirus 5
PCR	Polymerase Chain Reaction
qPCR	Quantitative Polymerase Chain Reaction
CFPHV	Chelonid Fibropapilloma-associated herpesvirus
ISH	In Situ Hybridization
UV	Ultraviolet

## References

[1] Jones K., Ariel E., Burgess G., Read M. (2016). A review of fibropapillomatosis in green turtles (*Chelonia mydas*). Vet. J..

[2] National Oceanic and Atmospheric Administration (NOAA) (2021). Fibropapillomatosis and Sea Turtles: Frequently Asked Questions. https://www.fisheries.noaa.gov/national/marine-life-distress/fibropapillomatosis-and-sea-turtles-frequently-asked-questions.

[3] Manes C., Pinton D., Canestrelli A., Capua I. (2022). Occurrence of Fibropapillomatosis in Green Turtles (*Chelonia mydas*) in Relation to Environmental Changes in Coastal Ecosystems in Texas and Florida: A Retrospective Study. Animals.

[4] Herbst H.H. (1994). Fibropapillomatosis of marine turtles. Annu. Rev. Fish Dis..

[5] Duffy D.J., Martindale M.Q. (2019). Perspectives on the expansion of human oncology and genomic approaches to sea turtles fibropapillomatosis. Commun. Biol..

[6] Work T.M., Dagenais J., Willimann A., Balazs G., Mansfield K., Ackermann M. (2020). Differences in antibody responses against Chelonid alphaherpesvirus 5 (ChHV5) suggest differences in virus biology in ChHV5-seropositive green turtles from Hawaii and Florida. J. Virol..

[7] Work T.M., Balazs G.H. (1999). Relating tumor score to hematology in green turtles with fibropapillomatosis in Hawaii. J. Wildl. Dis..

[8] Page-Karjian A., Norton T.M., Ritchie B., Brown C.A., Mader D.R. (2014). Factors influencing survivorship of rehabilitating green turtles (*Chelonia mydas*) with fibropapillomatosis. J. Zoo Wildl. Med..

[9] Whilde J., Mashkour N., Koda S.A., Eastman C.B., Thompson D., Burkhalter B., Frandsen H.R., Page A., Blackburn N.B., Jones K. (2024). International overview of sea turtle fibropapillomatosis: A survey of expert opinions and trends. Front. Cell Dev. Biol..

[10] Cazabon-Mannette M., Phillips A.C.N. (2017). Occurrence of fibropapilloma tumours on green sea turtles (*Chelonia mydas*) in Trinidad, West Indies. Living World J. Trinidad Tobago Field Nat. Club.

[11] Tripepi M., van Veghel I.J.R., Vreugdenhil A.D., Brunelli E. (2025). First report of fibropapillomatosis and critical habitat use in green sea turtles in Curaçao. Eur. Zool. J..

[12] Monezi T.A., Mehnert D.U., de Moura E.M., Müller N.M., Garrafa P., Matushima E.R., Werneck M.R., Borella M.I. (2016). Chelonid herpesvirus 5 in secretions and tumor tissues from green turtles (*Chelonia mydas*) from southeastern Brazil: A ten-year study. Vet. Microbiol..

[13] Lawrance M.F., Mansfield K.L., Sutton E., Savage A.E. (2018). Molecular evolution of fibropapilloma-associated herpesviruses infecting juvenile green and loggerhead sea turtles. Virology.

[14] James A., Page-Karjian A., Charles K.E., Edwards J., Gregory C.R., Cheetham S., Buter B.P., Marancik D.P. (2021). Chelonid alphaherpesvirus 5 prevalence and first confirmed case of sea turtle fibropapillomatosis in Grenada, West Indies. Animals.

[15] da Cruz K.P.P., Gattamorta M.A., Matushima E.R., Salvarani F.M. (2024). Fibropapillomatosis: A review of the disease with attention to the situation on the northern coast of Brazil. Animals.

[16] Lackovich J.K., Brown D.R., Homer B.L., Garber R.L., Mader D.R., Moretti R.H., Patterson A.D., Herbst L.H., Oros J., Jacobson E.R. (1999). Association of herpesvirus with fibropapillomatosis of the green turtle (*Chelonia mydas*) and the loggerhead turtle (*Caretta caretta*) in Florida. Dis. Aquat. Org..

[17] Quackenbush S.L., Casey R.N., Murcek R.J., Paul T.A., Work T.M., Limpus C.J., Chaves A., duToit L., Perez J.V., Aguirre A.A. (2001). Quantitative analysis of herpesvirus sequences from normal tissue and fibropapillomas of marine turtles using real-time PCR. Virology.

[18] Duarte A., Faísca P., Loureiro N.S., Rosado R., Gil S., Pereira N., Tavares L. (2012). First histological and virological report of fibropapilloma associated with herpesvirus in *Chelonia mydas* at Príncipe Island, West Africa. Arch. Virol..

[19] Vanstreels R.E.T., Durant A., Santos A.P., Santos R.G., Sarmiento A.M.S., Rossi S., Setim F.E., Gattamorta M.A., Matushima E.R., Mayorga L.F.S.P. (2023). Exploring the relationship between environmental drivers and the manifestation of fibropapillomatosis in green turtles (*Chelonia mydas*) in eastern Brazil. PLoS ONE.

[20] Reséndiz E., Fernández-Sanz H., Domínguez-Contreras J.F., Ramos-Díaz A.H., Mancini A., Zavala-Norzagaray A.A., Aguirre A.A. (2021). Molecular characterization of Chelonid alphaherpesvirus 5 in a black turtle. Animals.

[21] Espinoza J., Alfaro-Núñez A., Cedillo-Peláez C., Fernández-Sanz H., Mancini A., Zavala-Norzagaray A.A., Ley-Quiñonez C.P., López E.S., Garcia-Bereguiain M.A., Aguirre A.A. (2024). Epidemiology of marine turtle fibropapillomatosis and tumour-associated chelonid alphaherpesvirus 5. Vet. Res. Commun..

[22] Manes C., Carthy R.R., Hull V. (2023). A coupled human and natural systems framework to characterize emerging infectious diseases—The case of fibropapillomatosis in marine turtles. Animals.

[23] Valenzuela E., Bilimoria A., Ruggeri E., Vitales I., Penesso G., O’Pella J., van Veghel I., Vreugdenhil A., Ashley D.M., Tripepi M. (2026). Spatial differences in copper and lead levels in seagrasses and seaweeds from Curaçaoan bays. Caribb. J. Sci..

[24] Bastos K.V., Machado L.P., Joyeux J.C., Ferreira J.S., Militão F.P., Fernandes V.O., Santos R.G. (2022). Coastal degradation impacts on green turtle diet in southeastern Brazil. Sci. Total Environ..

[25] Keller J.M., Kucklick J.R., Harms C.A., McClellan-Green P.D. (2004). Organochlorine contaminants in sea turtles: Correlations between whole blood and fat. Environ. Toxicol. Chem..

[26] Perrault J.R., Stacy N.I., Lehner A.F., Mott C.R., Hirsch S., Gorham J.C., Buchweitz J.P., Bresette M.J., Walsh C.J. (2017). Potential effects of brevetoxins and toxic elements on various health variables in sea turtles after a red tide bloom event. Sci. Total Environ..

[27] Alfaro-Núñez A., Frost Bertelsen M., Bojesen A.M., Rasmussen I., Zepeda-Mendoza L., Tange Olsen M., Gilbert M.T.P. (2014). Global distribution of chelonid fibropapilloma-associated herpesvirus among clinically healthy sea turtles. BMC Evol. Biol..

[28] Aguirre A.A., Balazs G.H., Spraker T.R., Murakawa S.K.K., Zimmerman B. (2002). Pathology of oropharyngeal fibropapillomatosis in green turtles (*Chelonia mydas*). J. Aquat. Anim. Health.

[29] Manes C., Herren R.M., Page A., Dunlap F.D., Skibicki C.A., Rollinson Ramia D.R., Farrell J.A., Capua I., Carthy R.R., Duffy D.J. (2023). Green turtle fibropapillomatosis: Tumor morphology and growth rate in a rehabilitation setting. Vet. Sci..

[30] Work T.M., Balazs G.H., Rameyer R.A., Morris R.A. (2004). Retrospective pathology survey of green turtles (*Chelonia mydas*) with fibropapillomatosis in the Hawaiian Islands, 1993–2003. Dis. Aquat. Org..

[31] Okoh G.R., Horwood P.F., Whitmore D., Ariel E. (2021). Herpesviruses in reptiles. Front. Vet. Sci..

[32] Norton T.M., Jacobson E.R., Sundberg J.P. (1990). Cutaneous fibropapillomas and renal myxofibroma in a green turtle (*Chelonia mydas*). J. Wildl. Dis..

[33] Kang K.I., Torres-Velez F.J., Zhang J., Moore P.A., Moore D.P., Rivera S., Brown C.C. (2008). Localization of fibropapilloma-associated turtle herpesvirus in green turtles. J. Comp. Pathol..

[34] Muñoz Tenería F.A., Labrada-Martagón V., Herrera-Pavón R.L., Work T.M., González-Ballesteros E., Negrete-Philippe A.C., Maldonado-Saldaña G. (2022). Fibropapillomatosis Dynamics in Green Sea Turtles *Chelonia mydas* over 15 Years of Monitoring in Akumal Bay, Quintana Roo, Mexico. Dis. Aquat. Org..

[35] Robinson D.P., Jabado R.W., Rohner C.A., Pierce S.J., Hyland K.P., Baverstock W.R. (2017). Satellite tagging of rehabilitated green sea turtles (*Chelonia mydas*) from the United Arab Emirates, including the longest tracked journey for the species. PLoS ONE.

[36] Mashkour N., Jones K., Wirth W., Burgess G., Ariel E. (2021). The Concurrent Detection of Chelonid Alphaherpesvirus 5 and *Chelonia mydas* Papillomavirus 1 in Tumoured and Non-Tumoured Green Turtles. Animals.

[37] Labastida-Estrada E., Lugo-Trejo K.M., Islas-Villanueva V., Benítez-Villalobos F., Abreu-Grobois F.A., Oceguera-Figueroa A. (2026). Prevalence, etiology, and transmission of fibropapillomatosis in Olive Ridley turtles at a mass-nesting colony in the Mexican Pacific. PLoS ONE.

[38] Whitmore L., Yetsko K., Farrell J.A., Page-Karjian A., Daniel W., Shaver D.J., Frandsen H.R., Walker J.S., Crowder W., Bovery C. (2021). Evolutionary Comparisons of Chelonid Alphaherpesvirus 5 (ChHV5) Genomes from Fibropapillomatosis-Afflicted Green (*Chelonia mydas*), Olive Ridley (*Lepidochelys olivacea*) and Kemp’s Ridley (*Lepidochelys kempii*) Sea Turtles. Animals.

[39] Work T.M., Dagenais J., Weatherby T.M., Balazs G.H., Ackermann M. (2017). In vitro replication of Chelonid herpesvirus 5 in organotypic skin cultures from Hawaiian green turtles (*Chelonia mydas*). J. Virol..

[40] Farrell J.A., Yetsko K., Whitmore L., Whilde J., Eastman C.B., Ramia D.R., Thomas R., Linser P., Creer S., Burkhalter B. (2021). Environmental DNA monitoring of oncogenic viral shedding and genomic profiling of sea turtle fibropapillomatosis reveals unusual viral dynamics. Commun. Biol..

[41] Keller J.M., Balazs G.H., Nilsen F., Rice M., Work T.M., Jensen B.A. (2014). Investigating the potential role of persistent organic pollutants in Hawaiian green sea turtle fibropapillomatosis. Environ. Sci. Technol..

[42] da Silva C.C., Klein R.D., Barcarolli I.F., Bianchini A. (2016). Metal contamination as a possible etiology of fibropapillomatosis in juvenile female green sea turtles (*Chelonia mydas*) from the southern Atlantic Ocean. Aquat. Toxicol..

[43] Aguirre A.A., Lutz P.L. (2004). Marine turtles as sentinels of ecosystem health: Is fibropapillomatosis an indicator?. EcoHealth.

[44] Brooks D.E., Ginn P.E., Miller T.R., Bramson L., Jacobson E.R. (1994). Ocular fibropapillomas of green turtles (*Chelonia mydas*). Vet. Pathol..

[45] Rossi S., Zamana R., Andrade Santos P.P., Bomfim A., Farias D., Freire A., Oliveira R., Gattamorta M., Matushima E., Pires J.M.d.L. (2021). Visceral neoplasms and Chelonid alphaherpesvirus 5 in green turtles with fibropapillomatosis. Arch. Vet. Sci..

[46] Rodríguez C.E., Henao Duque A.M., Steinberg J., Woodburn D.B., Terio K., McAloose D., Leger J.S. (2018). Chelonia. Pathology of Zoo and Wildlife Animals.

[47] Work T.M., Rameyer R.A., Balazs G.H., Cray C., Chang S.P. (2001). Immune status of free-ranging green turtles with fibropapillomatosis from Hawaii. J. Wildl. Dis..

[48] Cray C., Varella R., Bossart G.D., Lutz P. (2001). Altered in vitro Immune Responses in Green Turtles (*Chelonia mydas*) with Fibropapillomatosis on JSTOR. J. Zoo Wildl. Med..

[49] Florida Fish and Wildlife Conservation Commission (2022). Fibropapillomatosis and Its Effect on Green Sea Turtles. https://myfwc.com/research/wildlife/sea-turtles/threats/fibropapillomatosis/.

[50] Saladin C., Freggi D. (2024). Recommendation of consensus definition of sea turtle fibropapillomatosis. Wildl. Lett..

[51] Pérez Y.A.A., Lima S.R., Martinez-Souza G., Gião T., Chenard G., Helayel M.J.A., Mársico E.T., da Silva K.V.G.C., de Alencar N.X. (2024). First case report of fibropapillomatosis tumor regression identified through photoidentification and histopathology in a *Chelonia mydas* in Itapirubá, Santa Catarina, Brazil. Open Vet. J..

[52] Jacobson E.R., Mansell J.L., Sundberg J.P., Hajjar L., Reichmann M.E., Ehrhart L.M., Walsh M., Murru F. (1989). Cutaneous fibropapillomas of green turtles (*Chelonia mydas*). J. Comp. Pathol..

[53] Roost T., Schies J.A., Girondot M., Robin J.P., Lelong P., Martin J., Siegwalt F., Jeantet L., Giraudeau M., Le Loch G. (2022). Fibropapillomatosis prevalence and distribution in immature green turtles (*Chelonia mydas*) in Martinique Island (Lesser Antilles). EcoHealth.

[54] Jacobson E.R., Buergelt C., Williams B., Harris R.K. (1991). Herpesvirus in cutaneous fibropapillomas of the green turtle *Chelonia mydas*. Dis. Aquat. Org..

[55] Jacobson E.R. (2007). Infectious Diseases and Pathology of Reptiles: Color Atlas and Text.

[56] Norton T.M., Walsh M.T., Miller R.E., Fowler M.E. (2012). Sea turtle rehabilitation. Fowler’s Zoo and Wild Animal Medicine, Current Therapy.

[57] Page-Karjian A., Perrault J.R., Zirkelbach B., Pescatore J., Riley R., Stadler M., Zachariah T.T., Marks W., Norton T.M. (2019). Tumor re-growth, case outcome, and tumor scoring systems in rehabilitated green turtles (*Chelonia mydas*) with fibropapillomatosis. Dis. Aquat. Org..

[58] Garefino L., Milton S.L. (2022). Influence of sunlight exposure on vitamin D concentrations in rehabilitating green sea turtles (*Chelonia mydas*). Animals.

[59] Robinson D.P., Hyland K., Beukes G., Vettan A., Mabadikate A., Jabado R.W., Rohner C.A., Pierce S.J., Baverstock W. (2021). Satellite tracking of rehabilitated sea turtles suggests a high rate of short-term survival following release. PLoS ONE.

[60] Baker L., Edwards W., Pike D.A. (2015). Sea Turtle Rehabilitation Success Increases with Body Size and Differs among Species. Wildl. Rehabil. Bull..

[61] Diggins R., Burrie R., Ariel E., Ridley J., Olsen J., Schultz S., Pettett-Willmett A., Hemming G., Lloyd J. (2022). A review of welfare indicators for sea turtles undergoing rehabilitation, with emphasis on environmental enrichment. Anim. Welf..

[62] Flint M., Patterson-Kane J.C., Limpus C.J., Mills P.C. (2010). Health surveillance of stranded green turtles in southern Queensland, Australia (2006–2009): An epidemiological analysis of causes of disease and mortality. EcoHealth.

[63] Flint J., Flint M., Limpus C.J., Mills P. (2017). Status of marine turtle rehabilitation in Queensland. PeerJ.

[64] Escobedo-Bonilla C.M., Quiros-Rojas N.M., Rudín-Salazar E. (2022). Rehabilitation of marine turtles and welfare improvement by application of environmental enrichment strategies. Animals.

